# A data set for mental and physical stress, loneliness, and life satisfaction before and during the COVID-19 pandemic and the role of working from home

**DOI:** 10.1186/s13104-025-07329-6

**Published:** 2025-07-01

**Authors:** Franziska Emmerich, Julia Junghans, Markus Zenger, Elmar Brähler, Yve Stöbel-Richter, Lisa Irmscher, Ernst Peter Richter, Hendrik Berth

**Affiliations:** 1 Division of Psychological and Social Medicine and Developmental Neurosciences, Research Group Applied Medical Psychology and Medical Sociology, Faculty of Medicine, TUD Dresden University of Technology, Fetscherstraße 74, 01307 Dresden, Germany; 2 Department of Differential and Personality Psychology, University of Applied Sciences Magdeburg-Stendal, Breitscheidstraße 2, 39114 Magdeburg, Germany; 3 Clinic and Polyclinic for Psychosomatic Medicine and Psychotherapy/Behavioral Medicine, University of Leipzig Medical Center, Liebigstraße 18, 04103 Leipzig, Germany; 4 Department of Psychosomatic Medicine and Psychotherapy, University Medical Center Mainz, Zahlbacher Str. 8, 55131 Mainz, Germany; 5 Department of Medical Psychology and Medical Sociology, University of Leipzig Medical Center, Philipp-Rosenthal-Strasse 55, 04103 Leipzig, Germany; 6 Faculty of Managerial and Cultural Studies, University of Applied Sciences Zittau/Görlitz, Furtstraße 3, 02826 Görlitz, Germany

**Keywords:** COVID-19, Corona pandemic, Saxon Longitudinal Studysleep disturbances, and heart proble, Mental well-being, Psychological strain, Physical health, Physical strain, Working from home, Home-based work

## Abstract

**Objectives:**

To examine mental and physical health, loneliness, and life satisfaction in East Germany, data were collected at four time points: in 2017/2018, 2019/2020, 2021, and 2022. Changes in data before and during the COVID-19 pandemic were determined using validated short scales. Additionally, a newly developed questionnaire was integrated in 2022 to depict working from home (WFH).

**Data description:**

The data set is part of the Saxon Longitudinal Study (SLS), first conducted in 1987 in the former German Democratic Republic and has been continued almost annually, consisting of 33 waves since then (*N* > 300). As an East German sample, the SLS offers a long-term perspective on psychological and physical health. The extent of mental health can be interpreted based on validated short scales D-Score (Distress Score), PHQ-4 (Patient Health Questionnaire-4), LS-S (Loneliness Scale), L-1 (Short Scale of Life Satisfaction), and a Corona Anxiety Scale. The physical strain can be interpreted based on the answers to the G-Score, SSS-8 (Somatic Symptom Scale), and an individual item on health status. The questionnaire on WFH allows comparisons between participants WFH and those who did not. This dataset will contribute to further research into events such as the COVID-19 pandemic and their impact.

## Objectives

The COVID-19 pandemic has been one of the most devastating global health crises of the 21st century[1]. Recent studies indicated a correlation between mental and physical well-being and the restrictions imposed by the German government to control the spread of the virus[2–6].

Moreover, the government encouraged businesses to utilize remote working options to reduce social interactions[7]. Before the pandemic in 2019, the proportion of workers WFH was 12.8%, followed by subsequent increases in the pandemic years (2020 = 21%, 2021 = 25%, 2022 = 24%) [8]. WFH was not only a preventive measure but also offered benefits such as flexible working hours and increased productivity[9]. However, recent international surveys revealed possible disadvantages like social isolation, work-life conflicts, or health problems[9].

Nevertheless, WFH has been very popular since the lockdown and could become more common in the future as pandemics seem to be more likely to occur. The SLS data collected before and during the COVID-19 pandemic in East Germany enables a detailed comparison of mental and physical well-being. Validated short scales ensured efficient data collection and facilitated the recording of specific health changes. An additional questionnaire was introduced in 2022 to characterize WFH due to its growing importance. The study by Emmerich et al. (2024), which was based on information from the SLS, reported no association between WFH and mental and physical health. The majority of participants WFH expressed satisfaction with WFH, indicating a willingness to continue working in this manner in the future [10].

The data set is of significant value to researchers, including those based outside of East Germany. It provides the opportunity to examine the circumstances under which comparable outcomes are observed in one’s own country. The results of this study facilitate the development of more effective strategies for responding to future public health crises, such as the pandemic caused by the SARS-CoV-2 virus.

## Data description

SLS data from 2017/2018 (wave 30), 2019/2020 (wave 31), 2021 (wave 32) to 2022 (wave 33), initially conceptualized as a result of research conducted on youth development in the German Democratic Republic (GDR) was investigated. The initial cohort consisted of 14-year-old 8th-grade East Germans (*N* = 1407), randomly selected from 72 different classes of 41 schools across the districts of Karl-Marx-Stadt and Leipzig in 1987. Gender distribution was comparable (female 47.2%). After the third wave (year: 1989), 41.7% (*N* = 587) of the respondents agreed to continue participating.[11].

Since 2002, validated and standardized questionnaires have been incorporated such as short scales investigating psychological (D-Score, PHQ-4, LS-S, L-1, and Corona Anxiety scale) and physical strain (G-Score, SSS-8, individual item on health status) before and during the COVID-19 pandemic. Attitudes towards WFH during the pandemic were examined since 2022.

The D-Score is a psychological stress indicator based on four key items: depression, meaninglessness of life, hopelessness, and fear of the future (waves 30,31,32,33)[12]. The PHQ-4 consists of two short scales, the PHQ-2, representing a depression scale, and the GAD-2 reflects an anxiety scale (waves 30,32,33)[13]. The LS-S was employed to assess feelings of exclusion, lack of sociability, and the experience of social isolation (waves 30,33)[14]. The single item L-1 measures life satisfaction (wave 30,31,32,33) [15]. The Corona Anxiety Scale is a tool designed to assess anxiety in the context of the COVID-19 pandemic (waves 32,33) [10].

The G-Score quantifies physical stress based on specific symptoms-nervousness, stomach problems, sleep disturbances, and heart problems-within the past 12 months (waves 30,31,32,33)[16]. The SSS-8 assesses the prevalence and severity of common somatic symptoms, including abdominal or digestive discomfort, back pain, and sleep disturbances, experienced within the past seven days (waves 30,33) [17]. The single-item health status serves as an indicator for the subjective health perception (waves 30,31,32,33) [18].

Attitudes towards WFH were evaluated using newly integrated questions into wave 33 (2022). Items were selected from the “Home Office Barometer 2020”[35], including productivity, loneliness, stress, childcare and partnership, future WFH, satisfaction, and working hours [19].

Data was gathered from almost 300 participants across each wave (wave 30 (2017/2018), *N* = 314; wave 31 (2019/2020), *N* = 323; wave 32 (2021), *N* = 321; wave 33 (2022), *N* = 319). Gender and age distribution among the waves were comparable (average birth year: 1973). Median age of respondents in wave 33 was 50 years, with the majority being in a partnership (> 80%).

**Table 1:** Overview of data sets

Label.	Name of data file/data set	File types (file extension)	Data repository and identifier (DOI or accession number)
*Data file 1*	Sächsische Längsschnittstudie Welle 30, 2017	IBM SPSS (.sav)	Leibniz Institute for the Social Sciences (gesis) 10.4232/1.13611 [20]
*Data file 2*	Sächsische Längsschnittstudie Welle 31, 2019	IBM SPSS (.sav)	Leibniz Institute for the Social Sciences (gesis) 10.4232/1.13612 [21]
*Data file 3*	Sächsische Längsschnittstudie Welle 32, 2020	IBM SPSS (.sav)	Leibniz Institute for the Social Sciences (gesis) 10.4232/1.13875 [22]
*Data file 4*	Sächsische Längsschnittstudie Welle 33, 2022	IBM SPSS (.sav)	Leibniz Institute for the Social Sciences (gesis) 10.4232/1.14147 [23]

## Limitations

The participants in the SLS represent a homogeneous age group (born in 1973), which precludes the possibility of generalizing to all age groups. All study participants were born in the federal state of Saxony in East Germany (former GDR). At the time of the survey, the majority of them were residing in Saxony. With regard to the level of education, the participants in the SLS exhibited a level of education that was slightly above the average for this age group, due to the design of the study. All participants had at least a 10th-grade qualification, while 38.7% had obtained a general university entrance qualification or higher.[11] The longitudinal study design restricted the number of socio-demographic characteristics surveyed. It was thus not feasible to incorporate the disparate levels of education, migration histories, age groups, or socialization experiences of the participants, who hailed from various regions of Germany, into the present study. A further limitation is the incomplete or interrupted follow-up of individuals over time, which could result in a loss of representativeness of the dynamic sample[24]. Another limitation are incomplete or interrupted follow-ups of individuals over time which could lead to a loss of representativeness of the dynamic sample[24]. The response rate decreased over the years (*N* = 587 in 1989 compared to *N* = 319 in 2022). In addition, the sample is limited as some participants did not take part in all surveys throughout[11].

The short scales used in this study depicted psychological and physical stress, life satisfaction, and loneliness. Although these are validated scales, they are still concise screening instruments. Nevertheless, complex topics are assessed by using fewer questions, which can lead to bias. Additionally, the recently developed WFH questionnaire, which incorporates selected items from the Home Office Barometer, has yet to undergo validation[19].

## Data Availability

The analysis is based on the Saxon Longitudinal Study. The data as well as all questionnaires with their individual items of the Saxon Longitudinal Study are archived at the Leibniz Institute for the Social Sciences (gesis) and can be obtained for research purposes at https://search.gesis.org/research_data/ZA6248 (accessed on 13 June 2024),https://search.gesis.org/research_data/ZA6249 (accessed on 13 June 2024), https://search.gesis.org/research_data/ZA7841 (accessed on 13 June 2024),https://search.gesis.org/research_data/ZA7842 (accessed on 13 June2024). Data files and codebooks for each wave are provided in German language. English translations of the codebooks were made available online, they can be used to translate the data. The English codebooks can be accessed via the links provided in Table 1. Detailed information about the study in English is also available online: . Please see Table 1 and references [20, 21–23] for details and links to the data Codebooks for every date file are publicly accessible without restriction. They contain the full wording of all questions and response categories as well as the complete descriptive statistics for each item (frequencies, percentages, means, minimum value, maximum value, standard deviations). The data files (IBM SPSS) are not publicly available due to privacy and ethical restrictions. The data must be requested by email to dataservices@gesis.org with an ultra-brief description of the research purpose. The depositors of the data grant written permission. This access route was chosen for various reasons: The participants gave their written informed consent to participate in the study in 1989. They did not provide their written consent for their data to be shared publicly, as the internet did not yet exist. The anonymized data sets also contain confidential or sensitive data, for instance on income, electoral behavior, and medical data. This access way also avoids duplicate publications, and it can be ensured that the origin of the data and the founding institutions are correctly named. Controlled disclosure is intended to prevent the data from being published elsewhere on the internet. The Leibniz Institute for the Social Sciences (gesis) charges a service price per data order. This amount is exclusively for gesis. The service fee is not a profit for the supplier of the data. It is a processing fee charged by the repository.

## Abbreviations

GDR: German Democratic Republic
L-1: Short Scale of Life Satisfaction
LS-S: Loneliness Scale
PHQ-4: Patient Health Questionnaire-4
SLS: Saxon Longitudinal Study
SSS-8: Somatic Symptom Scale
WFH: Working from home
